# Uterus-targeted liposomes for preterm labor management: studies in pregnant mice

**DOI:** 10.1038/srep34710

**Published:** 2016-10-11

**Authors:** Jerrie S. Refuerzo, Fransisca Leonard, Nataliya Bulayeva, David Gorenstein, Giuseppe Chiossi, Alejandra Ontiveros, Monica Longo, Biana Godin

**Affiliations:** 1 Division of Maternal Fetal Medicine, Department of Obstetrics, Gynecology and Reproductive Sciences, University of Texas Health Science Center at Houston, Houston, Texas, USA; 2 Department of Nanomedicine, Houston Methodist Research Institute, Houston, Texas, USA; 3 Department of NanoMedicine and Biomedical Engineering, University of Texas Health Science Center at Houston, Houston, Texas, USA; 4 Division of Maternal Fetal Medicine, Department of Obstetrics and Gynecology at University of Texas Medical Branch at Galveston, Galveston, Texas, USA.

## Abstract

Preterm labor caused by uterine contractions is a major contributor to neonatal morbidity and mortality. Treatment intended to reduce uterine contractions include tocolytic agents, such as indomethacin. Unfortunately, clinically used tocolytics are frequently inefficient and cross the placenta causing fetal side effects. Here we show for the first time in obstetrics the use of a targeted nanoparticle directed to the pregnant uterus and loaded with a tocolytic for reducing its placental passage and sustaining its efficacy. Nanoliposomes encapsulating indomethacin and decorated with clinically used oxytocin receptor antagonist were designed and evaluated *in-vitro, ex-vivo* and *in-vivo*. The proposed approach resulted in targeting uterine cells *in-vitro*, inhibiting uterine contractions *ex-vivo*, while doubling uterine drug concentration, decreasing fetal levels, and maintaining the preterm birth rate *in vivo* in a pregnant mouse model. This promising approach opens new horizons for drug development in obstetrics that could greatly impact preterm birth, which currently has no successful treatments.

Prematurity is a leading cause of perinatal morbidity and mortality affecting 9.6% of births in the United States^1^ and an estimated 15 million births worldwide^2^. Preterm labor is defined as regular contractions of the uterus resulting in changes in the cervix prior to 37 weeks of pregnancy^3^. Though there are a multitude of etiologies for preterm labor, the actual cause in any individual might sometimes be unclear. Premature newborns are at increased risk for both acute and chronic health problems, and developmental deficiencies^4^.

Due to maternal medical conditions or complication of pregnancy, medications are frequently essential for the health of the pregnant mother and fetus(es) requiring ongoing or episodic treatment^5^,^6^. Fetal exposure to medications most commonly occurs when free unbound drug crosses the placenta^7^,^8^,^9^. Targeting therapeutics to the affected tissue and minimizing the circulating free drug fraction for placental passage can open new opportunities in the field of obstetrics^10^.

Nanomedicine is a multidisciplinary field of research, merging notions of medicine and nanotechnology with the overall goal of accurately fine-tuning the biological processes that occur on the micron and submicron scales^11^,^12^,^13^. One of the great promises of nanomedicine over the traditional molecular therapeutics is the ability to vector drugs preferentially to the affected loci, thus increasing the efficacy and reducing associated adverse reactions. Nanoliposomes (liposomes, LIP) have been clinically used for over two decades to enhance drug delivery to tumor and infected loci, thereby reducing side effects of chemotherapeutic and antimicrobial therapies^12^,^13^,^14^,^15^, LIP, phospholipid based nanovesicles^13^,^14^,^15^, are biodegradable and generally pose no concern for toxicity.

Indomethacin (IND) is a tocolytic agent from the non-steroidal anti-inflammatory drugs (NSAID) family, which acts by reducing prostaglandin production in the maternal uterus^16^,^17^,^18^,^19^,^20^,^21^. Unfortunately, IND freely crosses the placenta^22^ and is associated with antenatal closure of the ductus arteriosus^23^,^24^,^25^, oligohydramnios^26^,^27^, necrotizing enterocolitis^28^, and intraventricular hemorrhage^29^ both in human and animal models^30^,^31^,^32^. Although IND is considered an effective tocolytic medication clinically available in the US to delay delivery for 48 hours to achieve corticosteroid therapy, it, like other tocolytic therapies, has never been able to improve short and long term neonatal outcomes^18^,^33^,^34^,^35^,^36^.

IND has a rapid onset of action and short half-life allowing its effects to be easily measured *in vitro* and *in vivo* using animal models^37^. The beneficial and adverse effects of IND have been shown in a multitude of studies revealing comparable effects in both pregnant women and pregnant rodents including IND’s ability to: (1) reduce uterine contractions^38^, (2) prolong pregnancy in the setting of preterm labor^16^,^17^,^18^,^19^,^20^,^21^, (3) cross the placenta^22^, and (4) cause stricture of the ductus arteriosus (DA)^23^,^24^,^25^,^30^,^31^,^32^. These inherent pharmacodynamics and pharmacokinetic properties make IND a good drug to investigate the potential for liposomes (LIP) to reduce placental passage and prevent adverse fetal effects in pregnant mice, which can then be extrapolated to humans. Our collaborative team has recently shown that untargeted LIP carrying IND (LIP-IND) reduced the placental passage of the drug to the fetus^39^.

Here we show for the first time in the field of obstetrics that a nanoparticle specifically designed for targeting the pregnant uterus is capable of increasing the fraction of the drug available to its intended site of action, while decreasing fetal exposure to the drug. For this purpose, we have fabricated LIP-IND-ORA, a LIP loaded with IND and decorated with a clinically available oxytocin receptor antagonist^40^ (ORA) on its surface, as schematically presented in Fig. 1. Oxytocin receptors are abundantly expressed on the pregnant uterus. ORA, (Atosiban^®^, Sigma, USA), is a clinically used tocolytic drug in Europe^40^. The objective of this study was to determine the ability of LIP-IND-ORA to specifically direct the delivery of IND to the pregnant uterus, inhibit uterine contractions, and reduce preterm birth.

## Results

### Liposome design and fabrication

To achieve active targeting of the LIP-IND system to the uterus, we have conjugated clinically used ORA to the liposome’s surface. For this purpose, the liposomes were engineered to include phospholipids with a spacer and carboxylic group (PEG-DSPE), which can react with the amino group of the ORA using a post-insertion technique. Various concentrations of the constituents were tested. Among the evaluated systems, 3% PEG-DSPE was found to be the most efficient in ORA conjugation (51.8% conjugation efficiency as normalized to the molar concentration of PEG-DSPE). Total ORA density was calculated to be ~1983 molecules/liposome. The resulting LIP-IND-ORA nanoliposomes are 124.2 ± 0.7 nm in size and possess negative zeta potential of −21.2 ± 0.4 mV. Molar ratio of ORA was 1.5% and IND was 10% of total phospholipid molecules in the liposome. LIP-IND-ORA also had a 93% IND loading efficiency meaning for 0.45 mg IND/mL liposome used in the production, 0.42 mg were incorporated into the liposomes. The loading rate was 3.69% (36.9 μg IND loaded per mg of LIP-IND-ORA). The addition of ORA to LIP-IND did not alter the drug encapsulation and fluorescent properties of the nanoparticles.

### Targeting efficiency *in vitro*

**Figure 2:**
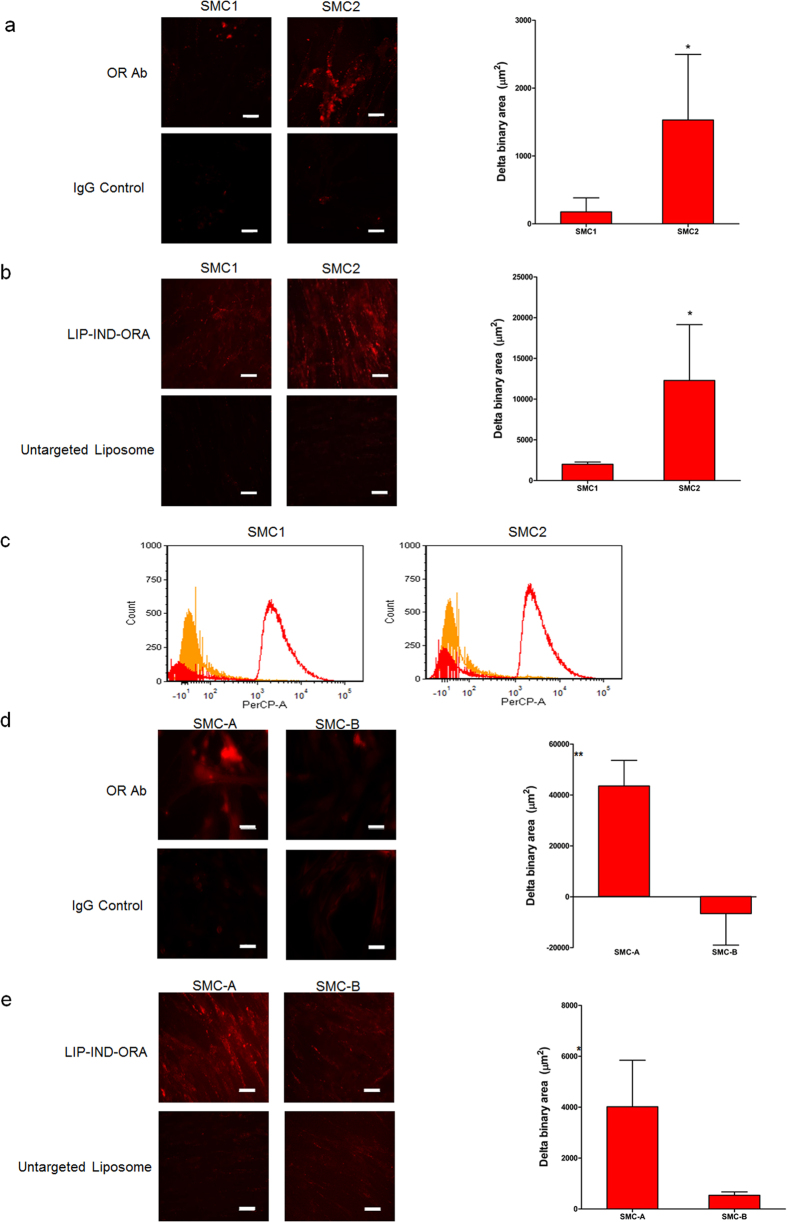
*In vitro* characterization of oxytocin receptor (OR) expression and correlated liposome targeting efficiency. The *in vitro* experiments were conducted in triplicates in primary smooth muscle cells SMC1 and SMC2 isolated from pregnant mice (**a–c**), and in human smooth muscle cell lines SMC-A and SMC-B (**d,e**). OR expression was verified by immunofluorescence staining with OR antibody (OR-Ab, red) and analyzed via confocal microscopy (**a,d**), using IgG staining as a negative control. Liposome targeting specificity was analyzed via confocal microscopy (**b,e**), as well as via flow cytometry (**c**). All the images were analyzed and quantified using NIS-elements. Mean ± SEM, n = 9 images per sample. Scale bar = 50 μm. *p-value < 0.05, **p-value < 0.01 compared to IgG control (**a,d**) or to untargeted liposome (**b,e**).

Expression level of oxytocin receptor (OR) was determined using immunofluorescence in smooth muscle cells (SMC) isolated from two pregnant mice (hence SMC1 and SMC2) (Fig. 2a). In both cases, the unspecific binding was tested using an isotypic IgG antibody. Both cells expressed OR, while SMC2 had on average four-times higher expression levels as compared to SMC1. The same trend is seen when the cells are incubated with fluorescently labeled targeted LIP-IND-ORA vs. untargeted liposomes (Fig. 2b). In both primary smooth muscle cells, LIP-IND-ORA enabled better attachment and retention when compared to untargeted liposomes. In terms of the targeting efficiency, LIP-IND-ORA attached on average eight-times more efficiently to SMC2 cells than to SMC1, which is in line with OR expression levels. The results were also confirmed via flow cytometry (Fig. 2c), where we found a significant increase in cells associated with LIP-IND-ORA as compared to untargeted liposome.

We have further tested an association of LIP-IND-ORA vs. untargeted liposomes with two human SMC (SMC-A and SMC-B). The results show significant increase of liposome accumulation when ORA targeting is in place by 5-fold and 23-fold, for SMC-A and SMC-B, respectively (Fig. 2d,e). Our data confirm that OR can be successfully targeted on murine and human uterine SMC using the proposed strategy.

### Biodistribution study *in vivo*

**Figure 3:**
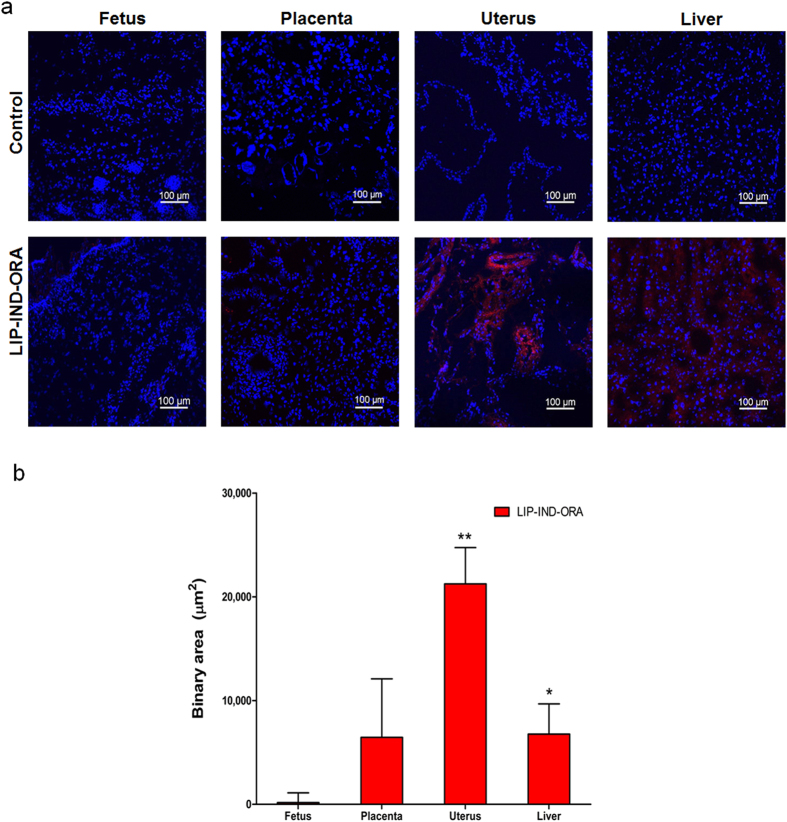
Biodistribution of LIP-IND-ORA components *in vivo* in pregnant mice. (**a**) Qualitative analysis of LIP-IND-ORA tissue distribution in the maternal uterus and liver, placenta and in the fetus of pregnant mice. Red: LIP-IND-ORA (lissamin rhodamin labeled phospholipid); Blue- DAPI, nuclear stain. (**b**) Quantification of the LIP-IND-ORA fluorescent signal in the tissues normalized to tissue auto-fluorescence using NIS elements. Quantitative biodistribution of liposome was obtained from at least 9 randomly selected fields per mouse of each organ. Mean ± SEM, n = 6. *p-value < 0.05, ^**^p-value < 0.01 to fetus.

To determine whether the targeted LIP-IND-ORA system would minimize the placental passage and deliver IND to the pregnant uterus, we evaluated the biodistribution of the systems *in vivo* by fluorescent microscopy of maternal and fetal tissues for tagged LIP-IND-ORA and analysis of IND by HPLC-MS/MS. Fluorescent microscopy was utilized to assess liposome localization to the uterine and fetal tissue. Since, it is known that nanoparticles tend to accumulate in the liver (the major organ of reticulo-endothelial system) the accumulations of the LIP-IND-ORA system and IND in the liver were also measured. Based on the fluorescent microscopy assessment of the tissues, the strongest signal of the system was in the uterus of the pregnant mice. LIP-IND-ORA was primarily confined within the uterus, minimally detected within the liver and placenta, and absent in the fetus as shown in Fig. 3a,b (see also Supplementary Fig. 1). Quantitatively, the binary area of a fluorescent signal associated with LIP-IND-ORA were more than three times higher in the uterus of animals given LIP-IND-ORA compared to liver, placenta and fetus, (uterus: 21,243 ± 3,502; liver: 6,768 ± 2,919; fetus: 168 ± 934 μm^2^, p < 0.05) [n = 6 per group, mean ± SEM].

**Figure 4:**
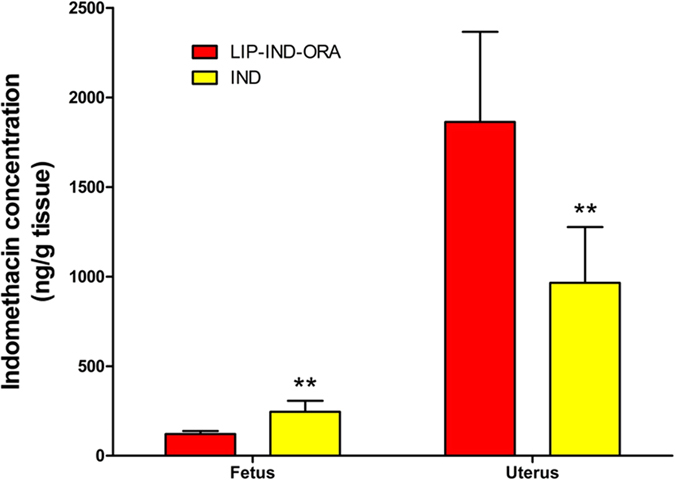
IND concentrations in the maternal uterus and fetus. To assess the shift in biodistribution in different formulations, IND was administered either in free form or within LIP-IND-ORA. IND concentrations in the maternal uterus and fetus were determined by LC-MS/MS analysis. Mean ± SEM, n = 6. ^**^p-value < 0.01 to the levels of IND when the drug was administered via LIP-IND-ORA.

Likewise, to assess reduction of placental passage of the drug from the LIP-IND-ORA system, the concentrations of IND were measured in the fetal tissue by liquid chromatography-mass spectrometry (LC-MS/MS). The concentration of IND in the uterine tissue was doubled in the pregnant mice receiving LIP-IND-ORA compared to IND alone, (LIP-IND-ORA: 1862.7 ± 503.3 ng/g vs. IND: 965.1 ± 311.7 ng/g, p = 0.006) as shown in Fig. 4. Moreover, there was a 2-fold reduction in levels of IND within the fetus of animals given LIP-IND-ORA compared to IND alone, (LIP-IND-ORA: 121.3 ± 16.8 ng/g vs. IND: 245.3 ± 61.7 ng/g, p = 0.002). Overall, the uterine to fetus IND concentration ratio was four fold higher for LIP-IND-ORA vs. IND. These findings demonstrated the targeting of the LIP-IND-ORA to the pregnant uterus tissue associated with reduction in the placental passage of IND.

### Uterine contractility *ex vivo*

**Figure 5:**
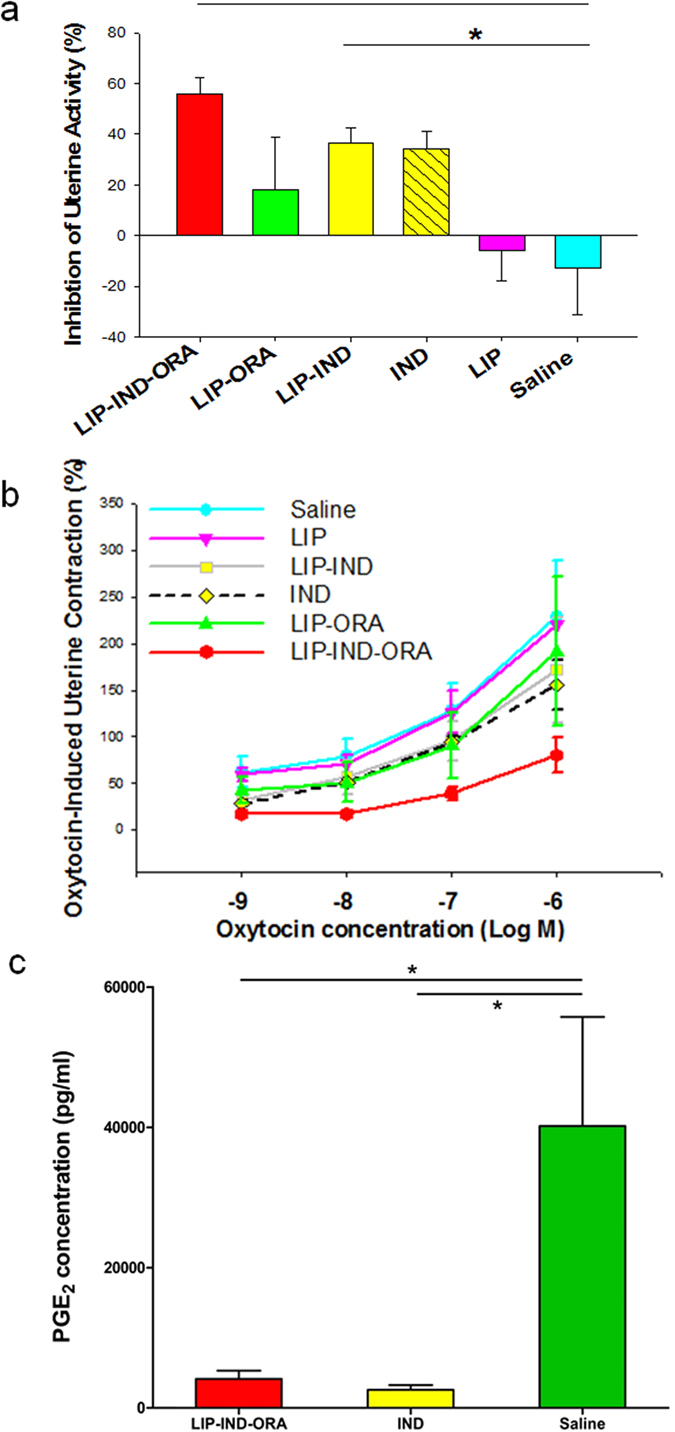
Inhibition of uterine contractility *ex vivo*. The efficacy of LIP-IND-ORA to inhibit contractility of uterus isolated from pregnant mice (GD 19) was examined. (**a**) Inhibition (%) of uterine contractions between LIP-IND-ORA, as compared to LIP-ORA, LIP-IND, IND, LIP and untreated control (saline, SAL) were determined in the absence or in the presence of various doses of oxytocin (an inducer of the uterine contractility) (**b**). (**c**) Prostaglandin E2 (PGE_2_) concentrations as determined by ELISA. Mean ± SEM. n = 6. *p-value < 0.05, ^**^p-value < 0.01 vs. untreated control.

We have further analyzed the efficacy of the proposed LIP-IND-ORA system in inhibiting the pregnant uterine contractility *ex vivo*. LIP-IND-ORA significantly increased the percent inhibition of uterine contractions compared to control (saline, SAL), (LIP-IND-ORA: 56.0 ± 6.4% vs. SAL: −12.8 ± 18.4%, p = 0.003) [n = 6 per group, mean ± SEM] as shown in Fig. 5a. Moreover, LIP-IND-ORA significantly increased the percent inhibition of uterine contractions compared to LIP, (LIP-IND-ORA, 56.0 ± 6.4, versus LIP −6.0 ± 11.8 p = 0.001). Interestingly, LIP-IND inhibited uterine contractions similarly to IND alone (36.8 ± 5.9% vs. 34.3 ± 6.6%, respectively), demonstrating the tocolytic efficacy of the drug while encapsulated in LIP. Finally, LIP-ORA showed no significant difference in uterine contractility (18.2 ± 20.4) compared to all the other groups. Representation of the myograph experiments for each drug and its effect on uterine contractility are given in Supplementary Fig. 2. Since saline functioned as the absence of a tocolytic agent, its exposure resulted in an increase in uterine contractions as designated by its negative value. The inhibition of uterine activity appeared to be more efficient with LIP-IND-ORA as compared to IND alone (IND: 34.3 ± 5.9). Further, to mimic the oxytocin-induced contractions in the uterus, a dose response curve to oxytocin (OXY) was also performed. In this setting LIP-IND-ORA showed a decreased OXY induced contraction curve compared to both IND and SAL at all doses of oxytocin (Fig. 5b).

To evaluate the pharmacological activity of IND encapsulated in the LIP-IND-ORA system, the levels of prostaglandin E2 (PGE_2)_ were determined in the uterus. PGE_2_ levels were significantly reduced in the uterus exposed to LIP-IND-ORA and IND compared to SAL, (4,127.9 ± 1,178.6, 2,587.4 ± 676.5 and 40,188.7 ± 15,555.6 pg/mL, respectively, p = 0.019) [n = 6 per group, mean ± SEM] as described in Fig. 5c. This illustrates that the encapsulation of IND within the targeted LIP, LIP-IND-ORA, does not alter the pharmacological activity of IND.

### Preterm birth *in vivo*

**Figure 6:**
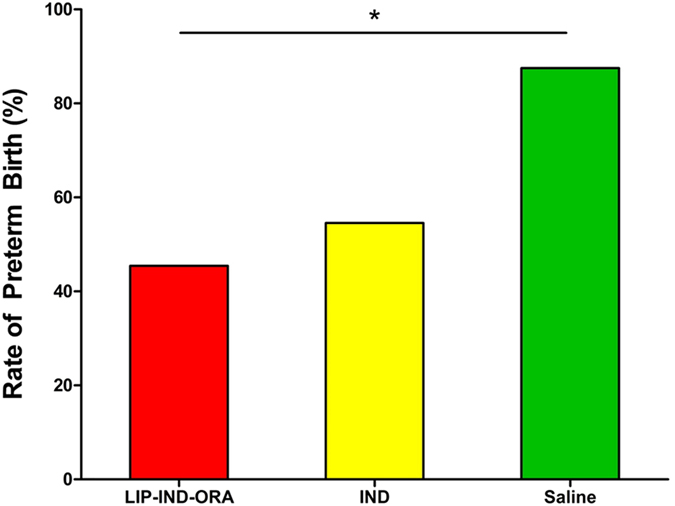
*In vivo* therapeutic efficacy in prevention of preterm birth. The efficacy of LIP-IND-ORA to prevent preterm birth was tested in an LPS-induced preterm pregnant mouse model. On GD 15, timed-pregnant CD1 mice were administered LPS (25 μg/kg) via intraperitoneal injection. Animals were randomly divided into 3 groups and given daily treatments (GD 15–17) via tail vein injection: IND (N = 11), LIP-IND-ORA (N = 13), and SAL (N = 8). IND concentrations were 1 mg/kg. Preterm birth rates (%) were compared between groups. *p-value = 0.029 compared to saline control.

LIP-IND-ORA significantly reduced the rate of lipopolysaccharide (LPS) induced preterm birth compared to SAL, (LIP-IND-ORA, n = 13: 46.2% vs. SAL, n = 8: 87.5%, p = 0.029) as shown in Fig. 6. There was a trend towards reduced rate of preterm birth by 15% with LIP-IND-ORA compared to IND alone (IND, n = 11: 54.5%), though this was not statistically significant. Additionally, although the length of pregnancy in hours was prolonged by 31% in mice treated with LIP-IND-ORA compared to IND and SAL, this was not statistically significant, (LIP-IND-ORA: 44.0 ± 4.5 h vs. IND: 30.0 ± 4.9 h vs. SAL: 17.5 ± 3.2 h, p = 0.076) [mean ± SEM].

## Discussion

Preterm birth continues to be the leading cause of perinatal mortality in the United States (US)^4^. The most recent National Vital Statistics Reports in 2015 report that death from prematurity (2.81 per 1000 births in 2013) is 2.3-fold higher than death from cancer (1.71 per 1000 adults from 2008–2012)^41^,^42^. Of the children that survive, 25–50% suffer long-term neurological impairment^4^. Based on a 2007 Institute of Medicine report, the annual financial burden from prematurity is estimated to be $26.2 billion or more than $51,000 per premature infant. Despite current use of prophylactic progesterone, pessaries, and cerclages^4^, there continues to be negligible improvements in the rate of preterm birth less than 34 weeks, 3.7% in 2006 to 3.4% in 2012^41^. Tocolytics remain the primary treatment for preterm labor to delay delivery^4^. The fundamental problems with tocolytic therapies are the inability to improve neonatal outcomes and potential adverse effects to the fetus^22^,^23^,^24^,^25^,^26^,^27^,^28^,^29^,^30^,^31^,^32^. Unfortunately, although highly demanded, there has been no significant improvement in tocolytic therapies for the past three decades, which can be ascribed to scant innovation in the field of drug therapies for preterm labor. In this manuscript, we pioneer the nanomedicine approach of targeted tocolytic therapy to address these fundamental problems unique to pregnancy by making therapeutics function better and safer for both mom and baby.

We were able to demonstrate successful delivery of indomethacin directly to the pregnant uterus with our customized liposome consistently across three approaches, *in vitro, in vivo* and *ex vivo*. A few months ago, King *et al*. have shown using fluorescent qualitative analysis that RGD-targeted nanoparticles can increase accumulate more in the mouse placenta than untargeted particles^43^. Our greatest achievement was reduction in the drug levels detected in the fetus, while maintaining indomethacin’s tocolytic efficacy. The targeted liposome significantly decreased prostaglandin levels in the uterus thereby inhibiting uterine contractions. This resulted in prolonging pregnancy by 31% and reducing the rate of preterm birth by 15% as compared to the free drug. To enable faster clinical translation of the proposed approach, we used a clinically used^40^ oxytocin receptor antagonist (ORA, or Atosiban) as our targeting element. In therapeutic concentration, ORA was shown to induce minimal adverse effects^40^. In the current study, the total administered dose of ORA was several times below the minimal therapeutic dose (see calculations in Supplementary information) and, consequently, ORA levels were not detectable in the maternal uterus and fetal tissue by LC-MS/MS. In this work we report for the first time in the field of obstetrics the use of targeted nanoparticles to improve high-risk pregnancy complicated with preterm labor. This promising approach opens new horizons for drug development in obstetrics that could greatly impact preterm birth, which currently has no successful treatments^36^.

## Methods

### Liposome design and fabrication

LIP, LIP-IND, LIP-ORA and LIP-IND-ORA were prepared by lipid hydration-extrusion method. First, the lipids were dissolved in 3 mL ethanol at the following concentrations: 9.6–12.2 mg soy bean phosphatidylcholine (Lipoid S100, Lipoid, Germany), 0–0.77 mg cholesterol (Sigma) and 1–3 mg DSPE-PEG(2000) Carboxylic Acid (1,2-distearoyl-sn-glycero-3-phosphoethanolamine-N-[carboxy(polyethylene glycol)-2000] (ammonium salt)) (Avanti, Alabama, USA). To fluorescently label LIP, fluorescent phospholipid Lissamine rhodamine B 1,2-dihexadecanoyl-sn-glycero-3-phosphoethanolamine, triethylammonium salt (rhodamine-DHPE, Invitrogen), 2% of the total lipid was incorporated to all liposome formulations. The incorporated Lissamine rhodamine B has excitation/emission maxima of 568/583 nm and was utilized for fluorescence microscopy and flow cytometry analysis. 0.45 mg of IND (Sigma), which corresponds to 10% of phospholipids molar content, was added to the above ethanolic mixture for LIP-IND and LIP-IND-ORA formulations. A thin film was formed by evaporating the solvent for 30 minutes (min), 41 °C at 150 rpm using rotary evaporator (Rotavapor, Buchi, Switzerland). The film was rehydrated with 1 mL PBS pH 7.2. Liposomes were extruded 10 times using each of the following 800-, 400-, and 200-nm Nuclepore Track-Etch Membrane (Whatman) filters with Lipex Biomembrane extruder. The resulting liposomes were ultracentrifuged (60,000 × g, 2 hours [h]) using Solvall WX ultra series ultracentrifuge (Thermo Scientific). Supernatant was removed and the LIP and LIP-IND were resuspended with 1 mL PBS.

ORA-NHS was prepared for conjugation with liposome by adding 1.9 mg EDC (1-ethyl-3-[3-dimethylaminopropyl]carbodiimide) (Life technologies) and 2.9 mg NHS (N-hydroxysuccinimide) to each mg ORA in MES buffer and incubated in rotator at room temperature (RT) for at least 15 min. For LIP-ORA and LIP-IND-ORA preparation, the systems were resuspended with 1 mL MES (2-[morpholino]ethanesulfonic acid) buffer containing ORA-NHS equivalent to 0.35 mg ORA weight. Conjugation was conducted at RT overnight, and unbound ORA were washed from liposome by ultracentrifugation (60,000 × g, 2 h).

The size and zeta potential of the liposomes were assessed by dynamic light scattering using Zetasizer (Malvern, Worcestershire, UK). Five separately prepared batches of each formulation were analyzed in triplicates each. The morphology and structure of LIP were observed by scanning electron microscopy as previously described^39^.

The levels of IND in the LIP were assessed using high-performance liquid chromatography (HPLC). Supernatant from ultracentrifugation after conjugation was used for measurement of unbound ORA concentration to determine the ORA conjugation efficiency indirectly. An aliquot of LIP-ORA and LIP-IND-ORA were dissolved in ethanol and used for direct measurement of conjugated ORA.

IND was analyzed by isocratic detection using ultraviolet diode-array HPLC system Column Hitachi Elite LaChrom, Column oven L-2300, Autosampler L-2200, Diode Array Detector L-2455, Pump L-2130 Hitachi D-2000 Elite v3.0 software. Kinetex 2.6 m XB-C18 100 Ǻ (100 × 4.6 mm; Phenomenex) column was used for the separation at 237 nm. Chromatography was performed using an isocratic elution with mobile phase composed of 0.2% phosphoric acid in acetonitrile at a flow rate of 0.6 mL/min with average retention time of 7.2 min.

### Animals

Pregnant female (strain CD1, stock N 022) were purchased form Charles River. For the targeting efficiency study and the biodistribution study, mice were used at term gestation, gestation day 18 (GD 18). For the *ex-vitro* uterine contractility study pregnant mice were used at gestational day 19 (GD 19), just before mice are about to deliver and uterine contractility is maximal. For the preterm study, mice were obtained at mid gestation (GD 14). The animal care and experiments were in accordance with the protocol #HSC-AWC-13-154 approved in January 2014 by the University of Texas Health Science Center at Houston (UTHSC-H) Animal Welfare Committee (AMC). The mice were housed separately in temperature and humidity-controlled quarters with constant 12:12-hours light-dark cycles in the animal care facility at the (UTHSC-H).

### Targeting efficiency *in vitro*

To confirm the localization of the oxytocin receptor on uterine cells, pregnant mouse uterine cell lines were created from two timed-pregnant CD1 mice on gestational day (GD) 18^44^,^45^. These pregnant mice were not involved in any prior study. After CO_2_ inhalation euthanasia, laparotomy was performed and the pregnant uterus was retrieved and placed in Hank’s balanced salt solution. The uterine tissue was cut into 1–2 mm fragments with a razor then digested in 0.1% trypsin (Sigma, USA) and 0.1% deoxyribonuclease (Sigma, USA) for 30 min at 37 °C in shaker incubator, followed by 0.1% collagenase (Sigma, USA) for another 30 min. After filtering the tissue through gauze, the cells were washed then plated on collagen I-coated 75 mm flasks (BD Biosciences, USA) with RPMI 1640 media (Sigma, USA), 10% fetal bovine serum (FBS, Sigma, USA) and Penstrept (Sigma, USA). The media was changed daily until Day 4. In addition, biopsies were obtained from two women undergoing cesarean section to create a cell culture of uterine cells. Both were obtained from singleton, non-laboring pregnancies at 39 weeks of gestation (Human A is Hispanic, BMI 29 kg/m^2^ and Human B is Caucasian, BMI 33 kg/m^2^). Institutional review board was approved August 2014 at UTHSCH, #HSC-MS-14-0370.

The study of liposome attachment was conducted in triplicates, where the cells were seeded with a density of 2 × 10^5^ cells/mL and 0.5 mL/well in 8-well chamber slide. The cells were incubated at 37 °C overnight for cell attachment. 10 μL (12.2 mg/mL) of either targeted or non-targeted liposomes that were tagged with lissamine rhodamine were added to each well and gently shaken for a homogenous distribution in the well. The slides were incubated at 37 °C for 4 h to allow for interaction between cells and liposomes. After the incubation, the medium containing liposomes were discarded and the cells were washed twice with PBS. Cells were fixed afterwards with 4% paraformaldehyde in PBS for 30 min. Slide chambers were removed with the provided slide separator and the slides were mounted using Prolong Gold Antifade reagent (Life Technologies) and sealed with Cytoseal XYL (Thermo Scientific). Fluorescence signal from liposomes was detected using Nikon Eclipse Ti fluorescence microscope and analyzed using NIS Elements software. Nine randomly selected microscopy area were taken for quantification of OR and accumulating liposomes.

For flow cytometry analysis, the cells were detached from the flasks by using the cell dissociation buffer (Life Technologies). Medium was removed from the flask and the cell layer was washed with calcium-/magnesium-free PBS. After PBS removal, 2 mL of the cell dissociation buffer was added to the cells and the flask was incubated at 37 °C for 10 min. Cells that are still attached after the time were detached by firmly tapping the flask. The cells were gathered by addition of medium and counted. At least 2 × 10^5^ cells were incubated with targeted and non-targeted liposomes in concentration of 0.244 mg/well for 4 h at 37 °C. After incubation time, the medium containing liposomes were removed by centrifugation at 400 × g for 5 min. The cells were analyzed using BD FACS Fortessa (Becton Dickinson, San Jose, CA) detected in the PerCP channel, using untreated cells as control. The data was post-processed using FCS Express Flow 5 software.

### Biodistribution study *in vivo*

The concentration of IND delivered to the pregnant uterus from LIP-IND-ORA was compared to free IND using our established *in vivo* pregnant mouse model^39^. On GD 18, timed-pregnant CD1 mice (N = 6/group) were randomly allocated to receive either LIP-IND-ORA, IND or saline (SAL) via tail vein injection at a volume of 0.1 mL. When the drug was used (LIP-IND-ORA or IND), the dose of IND was 1 mg/kg (range 50–60 mg per animal based on maternal weight)^31^,^37^. The *in vivo* doses were maintained across the study. After 4 h, pregnant mice were sacrificed by CO_2_ inhalation, followed by laparotomy to retrieve maternal liver, uteri, placentas and fetuses. The onset of action of LIP-IND-ORA is unknown when administered intravenously. However, indomethacin’s onset of action is 2–3 h when given orally to humans and rodents^22^, and is 4–5 h when encapsulated with liposomes and administered via intraperitoneal^38^. Moreover, our prior study showed an increase of IND in the uterus and reduction in the fetus when encapsulated within LIP after 4 h following administration^39^. Based on these prior studies, 4 h was chosen as the period of exposure.

Liposome distribution was determined by immunofluorescence as previously described^39^. Briefly, tissue localization of LIP was qualitatively assessed using fluorescent microscopy identifying the absence or presence of LIP (tagged with fluorescent dye as previously described) within the liver, uterus, placenta and fetus. For this analysis, the excised tissues were placed in cryo-molds, embedded in the cryo-preserving media (OCT) and immediately frozen using liquid nitrogen. The blocks were stored in −80 °C until sectioning using cryo-microtome. During mounting on the slides, the tissue slices were stained with DAPI (4′,6-diamidino-2-phenylindole) fluorescent stain to identify nuclear structures of cells. Images were taken with the BX51 fluorescent microscope (Nikon, USA) using filters for DAPI and Cy3 at 100× magnification. Six animals per group were utilized in this study and their organs were sectioned and analyzed. Quantification was conducted in randomly selected area of at least 9 areas per animal to ensure the objectivity, while an image was chosen to represent the fluorescence signal^46^. Quantitative biodistribution of liposome was determined using an NIS elements image processing software (Nikon, USA)^46^. The binary area of a fluorescent signal reported in μm^2^ as mean ± sem.

The concentrations of indomethacin in IND and LIP-IND-ORA samples were determined by LC-MS/MS using multiple reactions monitoring assay with Phenacetin-ethoxy-D5 (Sigma, USA) as internal standard. Uterine and fetal tissues were homogenized in 1 mL of ice–cold methanol/water (7:3 v/v). Phenacetin (final concentration 20 ng/mL) was added to each sample before centrifugation at 15,000 rpm for 10 min. Supernatant was dried under nitrogen and reconstituted in 0.1% formic acid aqueous solution followed by protein precipitation with acetonitrile (1:2). Samples were centrifuged again, supernatant was transferred to vial and 10 μL was injected to Shimadzu triple quad 8040 MS connected to LC system. Indomethacin concentrations were determined in ng/mL against calibrators. Calibration curve was prepared by spiking in calibrator levels (15.6; 31.2; 62.4; 125; 250 and 500 ng/mL) in control tissue (no indomethacin) followed by extraction procedure identical to sample preparation. Method parameters were: LOD = 3.9 ng/mL (accuracy 76%, S/N > 3); LOQ = 15.6 ng/mL (accuracy 85%; CV < 10%; S/N > 10) with correlation coefficient for linear regression R^2^ = 0.992. Indomethacin concentrations were further normalized per tissue weight (ng/g) and reported as mean ± SEM.

### Uterine contractility *ex vivo*

Our established *ex vivo* pregnant mouse model of uterine contractility was used to measure the ability of LIP-IND-ORA to inhibit uterine contractions^47^,^48^,^49^. On GD19, timed-pregnant mice (N = 6) underwent CO_2_ inhalation euthanasia, the maternal uteri were excised and placed into Krebs physiological solution. Uterine ring segments, 4 mm in width, were cut, and the fetuses and placentas were gently removed. The uterine rings were positioned between tungsten-wire (250 μm in diameter) stirrups and placed in an organ chamber containing 10 mL Krebs buffer, bubbled with 5% carbon dioxide in 95% oxygen maintained at constant temperature and pH (37 °C, pH 7.4). Passive tension was gradually applied to the optimal level of 1 g during an equilibration period of 60 min. Once the uterine tissue contracted spontaneously, uterine rings were then incubated with either: LIP-IND-ORA (10^−5^ mol/L), LIP-ORA, LIP-IND (10^−5^ mol/L), IND (10^−5^ mol/L), LIP and saline (SAL) as control for 40 min. After incubation of the study drugs, dose response curves to oxytocin (OXY) were obtained (10^−9^mol/L to 10^−6^mol/L; 20 min between OXY doses) to produce increased stable uterine contractions. The final concentration of IND administered into the organ chamber is equal to the concentration of IND (1 mg/kg) in LIP-IND and LIP-IND-ORA. To confirm tissue viability, potassium chloride (KCL 60 mmol/L) was added in each chamber at the end of the experiment. IND and OXY dose concentrations were based from previous uterine contractility studies^50^,^51^,^52^. OXY and IND (Sigma, USA) were dissolved in water and ethanol respectively. The final concentration of ethanol in the organ chamber solution (1.3 × 10^−4^mol/L) was 130 times inferior to a plasma concentration that could possibly account for a tocolytic effect^53^.

Changes in isometric tension were recorded with isometric force transducers (Harvard Apparatus, South Natik, MA) connected and stored to an online computer with data acquisition software (WINDAQ-200; DATAQ). The data was acquired and analyzed using Windaq data acquisition system (Dataq Instruments Inc, Akron, OH). Spontaneous contractile activity for each uterine ring was analyzed as an integral activity over 40 min before (basal activity) and after application of each study drug. Baseline activity was defined as the integral activity over the 40 min following stabilization of uterine contractions. The effect of the IND alone and LIP-IND-ORA were determined by calculating the integral activity expressed as a percent change from the baseline integral activity. OXY induced contractile response was expressed as an integral activity over 20 min of each OXY dose and the baseline uterine contractility^54^,^55^,^56^. The percent inhibition of uterine contractions and the dose response curve to oxytocin were calculated using software (Sigma Plot and GraphPad Prism, version 3.00 for Windows; GraphPad Software, San Diego, CA). Data was expressed as mean ± SEM.

Since the pharmacological action of IND involves the inhibition of prostaglandin production by cyclooxygenase, prostaglandin E_2_ (PGE_2_) levels were measured in uterine tissue using ELISA (ADI-901-001, Enzo Life Science, New York, USA). Another set of pregnant CD1 mice at GD 19 (n = 5) was used to evaluate uterus PGE_2_ levels. The uterus from the pregnant mice was obtained as described previously and incubated with LIP-IND-ORA, IND or saline as control for 40 min. The 40 min exposure time was chosen since this was the same length of time the uterine tissue was exposed to LIP-IND-ORA or IND in the uterine contractility experiments. PGE_2_ levels in each sample were then determined based on the instructions in the ELISA kit (ADI-901-001, Enzo Life Science, New York, USA). The concentration of PGE_2_ were expressed as pg/mL and reported as the mean ± SEM.

### Preterm birth *in vivo*

Our established *in vivo* preterm pregnant mouse model was used to test the ability of LIP-IND-ORA to prevent preterm birth. LPS is a commonly used model to induce labor in murine models^31^,^37^. Prior studies have demonstrated that when pregnant rodents are exposed to 50 μg/kg of LPS, preterm labor is induced in 90% of animals^31^. IND reduces the preterm birth rate in pregnant mice exposed to LPS by as much as 60–70%^31^,^37^. On GD 15, timed-pregnant CD1 mice received LPS (L2880, from Escherichia coli 055:B5, Sigma, USA) (25 μg/kg) via intraperitoneal injection. Animals were randomly divided into 3 groups and given daily treatments (GD15-17) via tail vein injection (100 μl volume) according to group randomization: IND (N = 11), LIP-IND-ORA (N = 13), and SAL (N = 8). IND concentrations for both IND and LIP-IND-ORA were again 1 mg/kg (range 50–60 mg per animal based on maternal weight) based on prior studies^31^,^37^. Since the LIP-IND-ORA solution color was visibly pink and due to limited resources to create a placebo, the randomization was not blinded. Each day after the LPS administration, the preterm birth rate was determined as the number of pregnant mice spontaneously delivering prior to GD18. Animals were single housed and were monitored continuously by direct observation and by video camera for the duration of the treatment period to confirm timing of delivery. The rate of preterm birth was expressed as percent of preterm delivery and length of pregnancy was expressed in hours after LPS administration. Results were reported as median ± sem.

A sample size calculation was performed based on prior experiments involving IND in the prevention of preterm birth in pregnant rodents. Mice treated with IND had a 30% rate of preterm birth compared to 90% in control^31^,^37^. Based on an effect of 33% with an α of 0.05 (2-tailed) and β of 0.80, we determined that 7 maternal mice were needed in each group.

### Statistical Analysis

Differences in isometric tensions of myometrium, IND concentrations in the uterus and fetus, PGE_2_ concentrations in the uterus, preterm birth rates and length of pregnancy between groups were analyzed using one-way analysis of variance with Tukey post hoc test. STATA software (version 12.1) were used and a P value < 0.05 was considered significant.

## Additional Information

**How to cite this article**: Refuerzo, J. S. *et al*. Uterus-targeted liposomes for preterm labor management: studies in pregnant mice. *Sci. Rep.* **6**, 34710; doi: 10.1038/srep34710 (2016).

## Supplementary Material

Supplementary Information

## Figures and Tables

**Figure 1:**
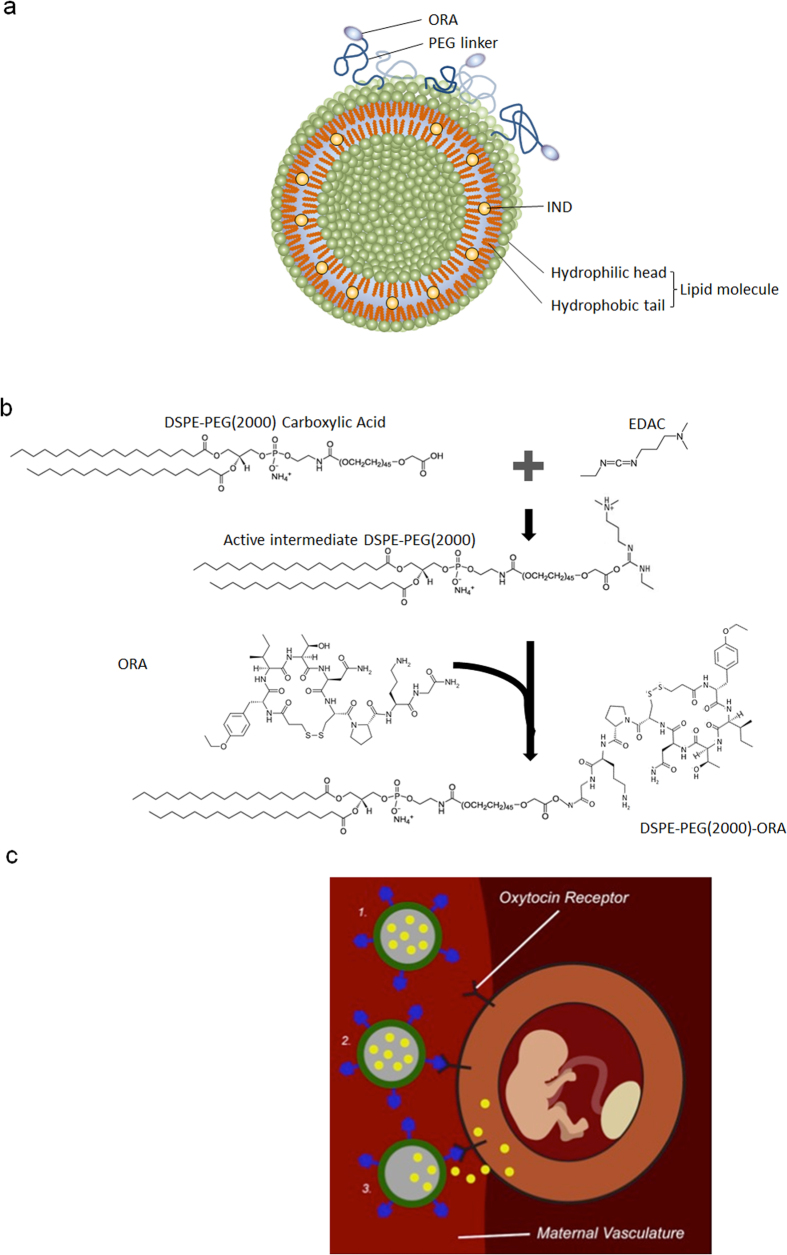
Schematic presentation of the LIP-IND-ORA design. (**a**) Illustration of LIP-IND-ORA structure and (**b**) Schematic of ORA conjugation to the LIP membrane. (**c**) Schematics of the LIP-IND-ORA mechanism of action: (1–2) binding to the oxytocin receptor expressed on the pregnant uterus and directing IND (yellow) specifically to the uterus (3) thereby improving the tocolytic efficacy of indomethacin while reducing its placental passage.
