# Supplementary material for: Uterus-targeted liposomes for preterm labor management: studies in pregnant mice
Source: Sci Rep. 2016 Oct 11;6:34710. doi: 10.1038/srep34710 (PMC5057095; doi:10.1038/srep34710)
Supplement: Supplementary Information [file srep34710-s1.pdf]

SUPPLEMENTARY INFORMATION

Uterus-targeted liposomes for preterm labor management: studies in pregnant mice

Jerrie S. REFUERZO, M.D.[1*]
Fransisca LEONARD, Ph.D. [2*]
Nataliya BULAYEVA, Ph.D [3]
David GORENSTEIN, Ph.D [3]
Giuseppe CHIOSSI, M.D.[4]
Alejandra ONTIVEROS, M.D. [1]
Monica LONGO,M.D., Ph.D [1#]
Biana GODIN, M.Sc.,Pharm. Ph. D. [2#]

[1] Division of Maternal Fetal Medicine, Department of Obstetrics, Gynecology and Reproductive Sciences, University of Texas Health Science Center at Houston, Houston, Texas, [2] Department of Nanomedicine, Houston Methodist Research Institute, Houston, Texas, [3] Department of NanoMedicine and Biomedical Engineering, University of Texas Health Science Center at Houston, Houston, Texas and [4]Division of Maternal Fetal Medicine, Department of Obstetrics and Gynecology at University of Texas Medical Branch at Galveston, Galveston, Texas.

[*]Shared first authorship
[#]Shared last authorship

Calculation of ORA concentration in LIP-IND-ORA as compared to the human dose

The dose of ORA was 35.8 µg/ mouse (~50g) or approximately 7mg/kg, which is, based on the accepted NIH conversion factors https://ncifrederick.cancer.gov/lasp/acuc/frederick/Media/Documents/ACUC42.pdf equivalent to human dose of 0.58 mg/kg.

In humans, ORA (Atosiban®) is given as an infusion in three steps: Initial loading dose of 6.75mg, followed by 300 µg/min for 3 hours (54mg) and up to 45 hours subsequent intravenous infusion at 100 µg/min. So the overall dose is in the range of 70-330mg/person or (considering the average weight of 60kg) 1.2-5.5 mg/kg. Thus, the given dose of ORA is below the physiologically relevant concentrations of the drug.

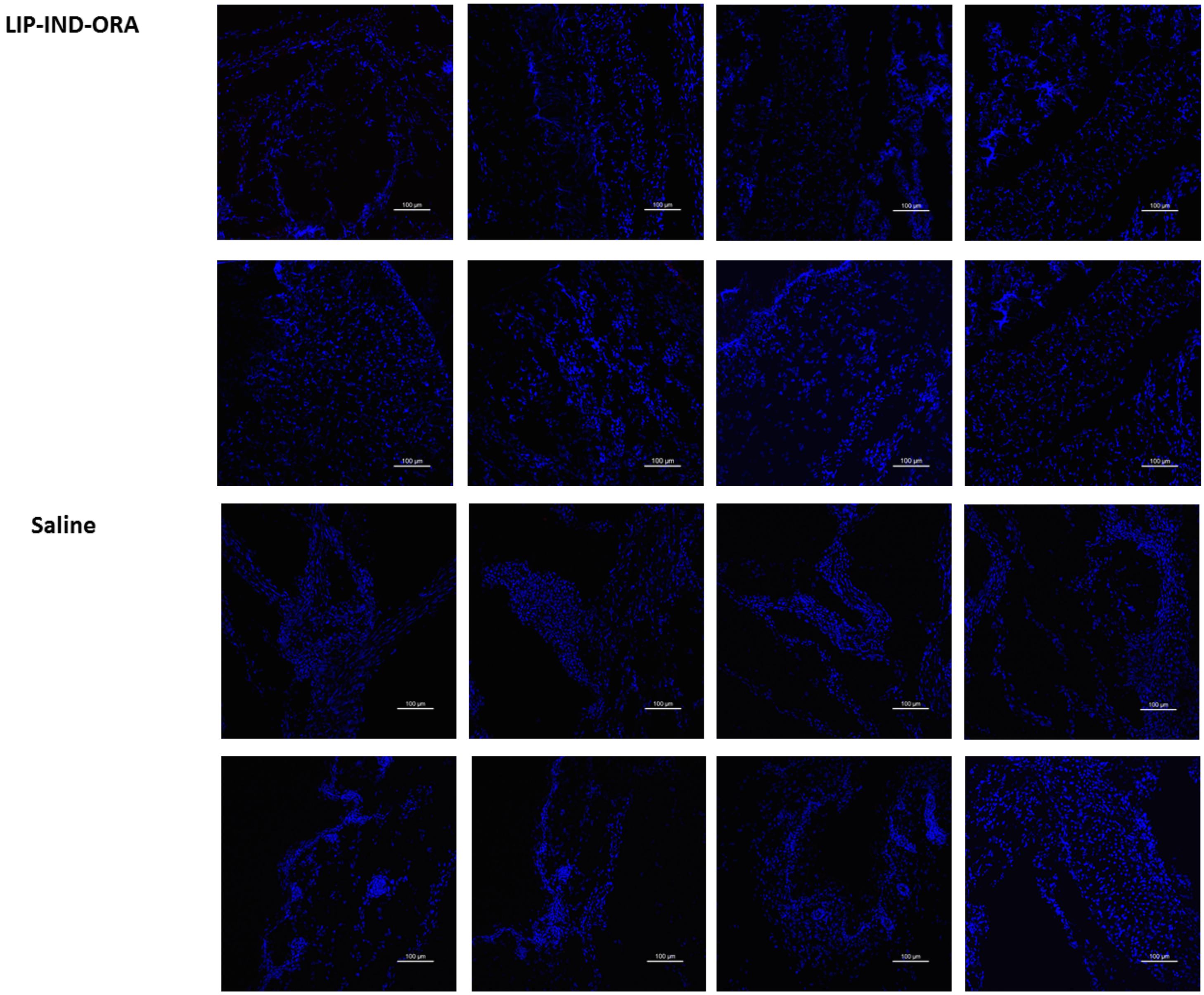

Supplementary figure 1: Additional images of fetuses of mice injected with LIP-IND-ORA vs. SAL. LIP-IND-ORA were fluorescently labeled (red tag) with lissamin rhodamin, and the samples were counterstained with DAPI. The absence of red fluorescence proves the lack of penetration of the liposomes to the fetus.

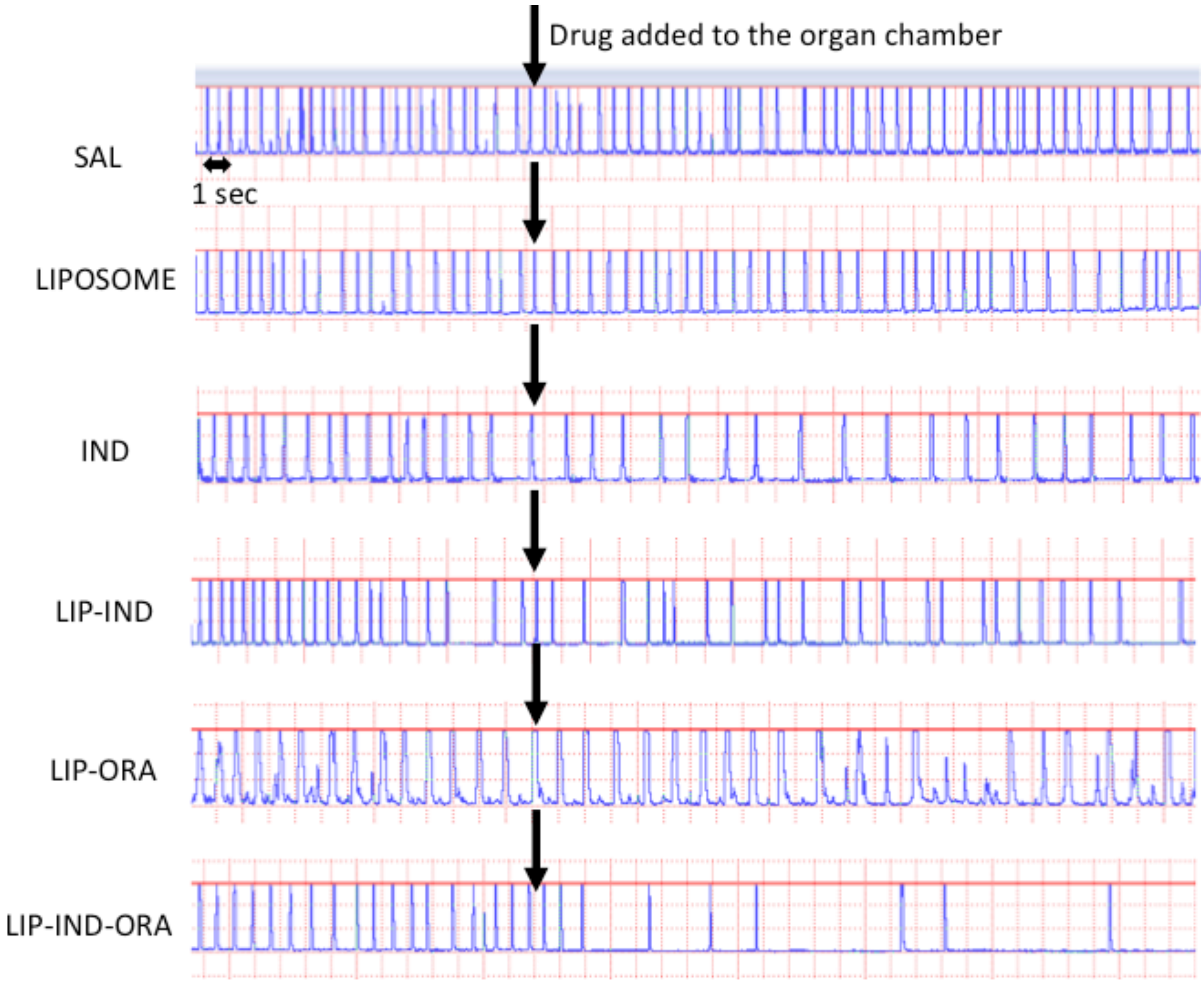

Supplementary Figure 2: Representative myographs from ex vivo contractility experiments.
